# An nhr-85::GFP::AID*::3xFLAG knock-in allele for investigation of molting and oscillatory gene regulation

**DOI:** 10.17912/micropub.biology.000993

**Published:** 2023-10-18

**Authors:** Krista M. Myles, John C. Clancy, Londen C. Johnson, Guinevere Ashley, Jesus Manzano, James Matthew Ragle, Jordan D. Ward

**Affiliations:** 1 Department of Molecular, Cell, and Developmental Biology, University of California, Santa Cruz, Santa Cruz, California, United States

## Abstract

*C. elegans* NHR-85 is a poorly characterized nuclear hormone receptor transcription factor with an emerging role in regulating microRNA expression to control developmental timing. We generated the first NHR-85 translational fusion by knocking a GFP::AID*::3xFLAG cassette into the endogenous locus to tag all known isoforms. nhr-85 ::GFP::AID*::3xFLAG animals have wild-type broodsizes and NHR-85 ::GFP peaks in expression at the start of the L4 stage in epithelial cells. NHR-85 is not expressed in the germline, suggesting that while it might cooperate with the NHR-23 transcription factor to control microRNA expression, NHR-23 promotes spermatogenesis independent of NHR-85 . This nhr-85 ::GFP::AID*::3xFLAG strain will be a valuable resource for studying when and where NHR-85 acts to promote developmental timing.

**Figure 1. NHR-85 ::GFP is expressed in epithelial cells but not the germline:**
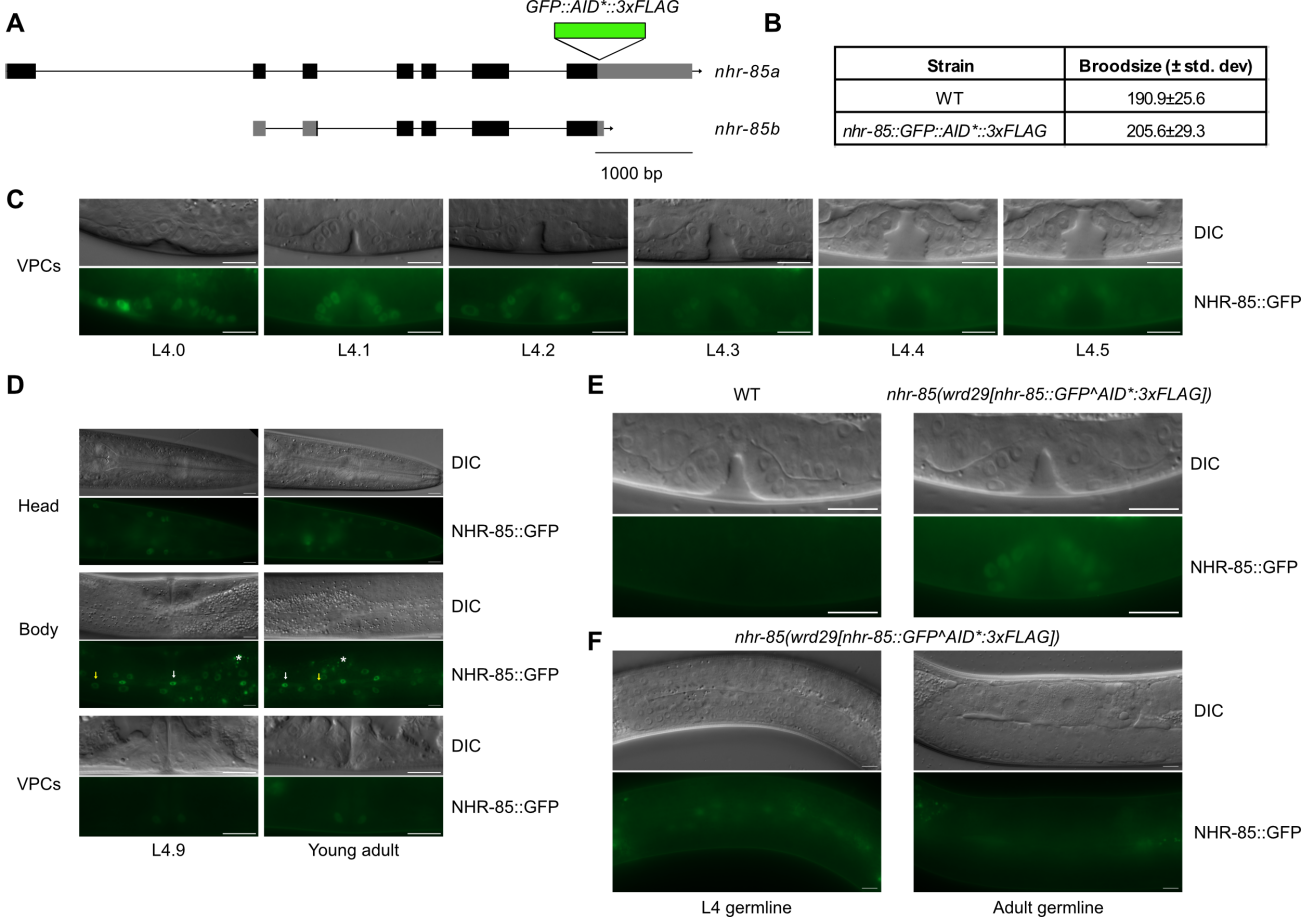
(A) Schematic of the *nhr-85* gene with location of the endogenous GFP::AID*::3xFLAG knock-in. Black rectangles are coding exons, gray rectangles are the 5’ and 3’ untranslated regions, and the arrow indicates the direction of the gene and position of the introns. (B) Brood size analysis of wild type and nhr-85::GFP::AID*::3xFLAG animals. n=11 for N2 (WT) and n=13 for JDW628 ( nhr-85::GFP::AID*::3xFLAG ). NHR-85::GFP expression in vulval precursor cells from L4.0-L4.5 (C) and in L4.9 and young adult animals (D). NHR-85::GFP expression in head epithelial cells and in hypodermal and seam cells in the animal body. Asterisks indicate examples of gut granule autofluorescence, white arrows point to representative seam cell nuclei, yellow arrows point to representative hypodermal nuclei. (E) The GFP signal in L4.2 vulval precursor cells is specific to nhr-85::GFP::AID*::3xFLAG animals and is not observed in wild-type controls. (F) NHR-85::GFP is not detectably expressed in L4 or adult germlines. Scale bars=10 µm in C-F. All images are representative of twenty animals examined over two independent experiments.

## Description

Molting is the process by which animals generate a new exoskeleton and shed their old one. Nematodes have a collagenous exoskeleton (cuticle) that is replaced at the end of each of four larval stages (Lažetić & Fay, 2017). The shedding and replacement of this cuticle is thought to be coordinated by a recently discovered, large-scale genetic oscillation in which ~20% of genes peak one time during each larval stage (Hendriks et al., 2014; Meeuse et al., 2020; Tsiairis & Großhans, 2021). The homologs of several mammalian circadian rhythm regulators such as Per and RORα ( LIN-42 and NHR-23 in *C. elegans* ) regulate *C. elegans* molting (Jeon et al., 1999; Kostrouchova et al., 1998, 2001; Monsalve et al., 2011) . NHR–85, the *C. elegans* homolog of another mammalian circadian rhythm regulator (Rev-ERBα), is a poorly-characterized nuclear hormone receptor (NHR) transcription factor implicated in molting (Gissendanner et al., 2004) . To gain insight into endogenous NHR-85 expression we used CRISPR/Cas9-mediated genome editing to insert a GFP::AID*::3xFLAG tag into the 3’ end of the gene to produce a C-terminal translational fusion to all predicted isoforms ( Figure 1A ). *nhr-85* RNAi was reported to cause an egg-laying defect (Gissendanner et al., 2004) . To test whether the tag disrupted NHR-85 function, we monitored nhr-85 ::GFP::AID*::3xFLAG egg laying in a broodsize assay and found that the strain had wild-type fecundity with no obvious egg-laying defect ( Figure 1B ) .

Our nhr-85 ::GFP::AID*::3xFLAG strain has been used to analyze how NHR-85 cooperates with the NHR-23 transcription factor to control the expression of the *lin-4* microRNA (Kinney et al., 2023) . We were able to reproduce the peak in expression of NHR-85 ::GFP in vulval precursor cells at L4.0 and L4.9 ( Figure 1C,D ). We also observed the reported expression in hypodermal cells as well as in seam cells ( Figure 1D ). We confirmed that the expression was specific to the nhr-85 ::GFP::AID*::3xFLAG strain, as no nuclear GFP signal was observed in wild-type animals ( Figure 1E ). NHR-85 ::GFP expression is nuclear and excluded from nucleoli ( Figure 1C,D ). Interestingly, we did not observe NHR-85::GFP expression in the L4 or adult germline ( Figure 1F ), and *nhr-85* null animals are viable and fertile (Kinney et al., 2023) . NHR-23 is expressed in the L4 and male germline and promotes spermatogenesis (Ragle et al., 2020, 2022) . These data suggest that while NHR-23 and NHR-85 cooperate in the soma to regulate gene expression, *nhr-23* has an *nhr-85 -* independent role in regulating spermatogenesis. Future use of this strain should allow for conditional, tissue-specific depletion to gain insight into when and where NHR-85 acts to promote oscillatory gene expression.

## Methods

*C. elegans strains and culture*

*C. elegans* strains (see table in Reagents) were cultured as originally described (Brenner, 1974) , except worms were grown on MYOB instead of NGM. MYOB was made as previously described (Church et al., 1995) . Animals were cultured at 20°C for all assays, unless otherwise indicated. For general strain propagation, animals were grown at 15°C according to standard protocols. Brood sizes were performed as previously described (Ragle et al., 2022) .

*Strain generation*

Knock-ins were generated by the self-excising cassette (SEC) CRISPR method (Dickinson et al., 2015) . An nhr-85 ::GFP::AID*::3xFLAG repair template (pJW1804) containing an sgRNA targeting the 3’ end of *nhr-85* was generated by SapTrap (Schwartz & Jorgensen, 2016) . 5’ and 3’ homology arms were PCR amplified with oligos 3492+3493 and 3494+3495, respectively (see Reagents), and then purified with a Qiagen PCR purification kit. Oligos 3490+3491 were annealed and SapTrap cloned as previously described with pDD379 (backbone), the purified 5’ and 3’ homology arm PCRs, pJW1347 ( *30 amino acid (aa)* linker; NT slot), pDD363 ( *SEC-LoxP* ), pDD373 ( *GFP-C1* ), and pJW1759 ( TEV::AID*::3xFLAG for CT slot) to generate pJW1804 (Ashley et al. 2021; Schwartz and Jorgensen 2016; Dickinson et al. 2018) . pJW1804 was injected into EG9615 , which stably expresses Cas9, and knock-in animals were recovered and the SEC was excised by heat-shock as previously described to generate JDW115 (Dickinson et al., 2015; Schwartz et al., 2021) . Strain JDW628 was generated by outcrossing JDW115 animals four times to wild-type N2 animals. The loss of the *oxSi1091* Cas9 transgene was confirmed by genotyping with oligos 5934+5935 (detects unmodified locus) and 5237+5238 (detects Cas9 transgene in locus). Loss of the *unc-119 ( ed3 )* allele was confirmed by phenotyping. The nhr-85 ::GFP::AID*::3xFLAG insertion was genotyped with oligos 4932+4933+3380. Genotyping reactions were performed using a 63ºC annealing temperature.

*Microscopy*

Imaging was performed as previously described (Johnson et al., 2023) . Animals were synchronized by alkaline bleaching and released on MYOB before harvesting at the indicated developmental timepoints. Animals were picked into a 15 µl drop of M9+5 mM levamisole on a glass slide with a 2% agarose pad and secured with a coverslip. Animals were imaged using a Plan-Apochromat 63×/1.4 Oil DIC lens on an AxioImager M2 microscope (Carl Zeiss Microscopy) equipped with a Colibri 7 LED light source and an Axiocam 506 mono camera. We used Fiji software (version: 2.0.0- rc-69/1.52p) to process images (Schindelin et al., 2012) . For the comparisons in the developmental timecourse or between strains, we set the exposure conditions to avoid pixel saturation of the brightest sample and kept equivalent exposure for imaging of the other samples.

## Reagents

**Table:** 

**Strain**	**Genotype**	**Available from**
N2	WT	CGC
EG9615	oxSi1091[Pmex-5::cas9(+ smu-2 introns):: tbb-2 3'UTR unc-119 +; *ttTi5605] II; unc-119( ed3 ) III	Prof. Erik Jorgensen
JDW114	nhr-85 (wrd28[nhr-85::GFP^SEC^AID*:3xFLAG]) I; oxSi1091[Pmex-5::cas9(+ smu-2 introns):: tbb-2 3'UTR unc-119 +; *ttTi5605] II; unc-119( ed3 ) III	Prof. Jordan Ward
JDW115	nhr-85 (wrd29[nhr-85::GFP^AID*:3xFLAG]) I; oxSi1091[Pmex-5::cas9(+ smu-2 introns):: tbb-2 3'UTR unc-119 +; *ttTi5605] II; unc-119( ed3 ) III	Prof. Jordan Ward
JDW628	nhr-85 (wrd29[nhr-85::GFP^AID*:3xFLAG]) I	Prof. Jordan Ward

**Table:** 

**Oligo number**	**Sequence (5' to 3')**	**Purpose**
3380	AAGAACGTGATGGTTTCCTGC	*nhr-85* knock-in genotyping
3490	GGCTGCTCTTCGTGGAACTCAGCGGGCAGTAGGTT	*nhr-85* SapTrap 5' arm (PAM mut)
3491	GGGTGCTCTTCGCGCTTCACTTAACGTTGTTGGCACAGGCGATACCTTCATC	*nhr-85* SapTrap 5' arm (PAM mut)
3492	GGCTGCTCTTCGACGGGCTGCTCTTCGACGTAATCAGTGATGATCTGGTTTCACGATCC	*nhr-85* SapTrap 3' arm
3493	GGGTGCTCTTCGTACTAGTGCACCTGGGAAGGAACT	*nhr-85* SapTrap 3' arm
3494	TTGGATTATTCGGAGAGTGTCGT	*nhr-85* 3' end sgRNA
3495	AACACGACACTCTCCGAATAATC	*nhr-85* 3' end sgRNA
4932	CCCACAGGACGCAAGTTTTG	*nhr-85* knock-in genotyping
4933	ACAGGCTTCACTGTACGCTTC	*nhr-85* knock-in genotyping
5234	ACGGATGCCTAGTTGCATTGA	Cas9 transgene genotyping
5235	GGCTTGTAACGCGGAATCAC	Cas9 transgene genotyping
5257	CTCGAGAAGATGGACGGAAC	Cas9 transgene genotyping
5238	CATTCCCTCGGTGACGTACT	Cas9 transgene genotyping

**Table:** 

**Plasmid**	**Reference**	**Description**
pDD363	Dickinson et al., 2018	LoxP-flanked SEC donor for the SapTrap cloning system
pDD372	Dickinson et al., 2018	Codon-optimized *GFP-C1 for SapTrap cloning*
pDD379	Dickinson et al., 2018	SapTrap destination vector for building repair templates. Contains *U6p::sgRNA(F+E)*
pJW1347	Ashley et al., 2021	*30 amino acid linker* for SapTrap CT slot
pJW1759	Ashley et al., 2021	TEV-AID*-3xFLAG for SapTrapNT slot
pJW1804	This study	nhr-85 3' CRISPR repair template - nhr-85::30xlinker::GFP::SEC+LoxP::TEV::AID*::3xFLAG
